# Essay on Cholera Infantum*We have been honored by a number of pamphlets from M. L. Knapp, M. D., on which as yet we have expressed no opinion. We propose now to let the Boston Journal speak for us.—Ed. Buff. Med. Jour.

**Published:** 1856-03

**Authors:** 


*Essay on Cholera Infantum.** By M. L. Knapp, M. D., late Professor of Materia Medica, and President of the College of Physicians and Surgeons of the University of Iowa; late Prof. of Midwifery and Diseases of Women and Children in Rush Med. College, &c. Cincinnati: H. W. Derby. 1855.

A pamphlet upon "Epidemic Cholera," by this writer, was noticed in our last volume (p. 264.) It has since been made quite evident by him, in an "Appendix" to a new issue of his paper, that our remarks were decidedly unpalatable, and we were therefore much surprised to find this "Essay" upon our table, especially as its author went out of his way to state, in the above-mentioned appendix, that "the opinion of that cloistered journalist (that's *us*) is perhaps of no consequence." Very likely—but why send us anything more so far above us? We really do not crave it. The above petulant remark rather amuses us than otherwise, and as much from its absurdity of diction as its unmeaningness and inapplicability.

In respect to the insane attempts of M. L. Knapp, M. D., to generalize a set of affections and refer them to one sole originating cause, merely because certain of the symptoms manifested in each are likewise found, at times, in all, we leave them, without comment, to the disposal of the common sense of the profession. At the rate this innovator goes on, we shall find *every* disease brought at last under the category *Scorbutus:* dysentery, it would seem, must come next; or possibly, common diarrhœa: we really should not be surprised if meningitis and apoplexy (scurvy things enough to deal with, to be sure) arose from a concealed scorbutic taint which thus strikingly manifests itself at the last: lemon and lime-luice, &c., will save such patients; let us have an "Essay" to show us the indications for their employment.

Our "cloistered opinion" being, however, once more called for, we can only refer to one or two points, when we shall turn with a sense of relief from these inane pages.

---

* We have been honored by a number of pamphlets from M. L. Knapp, M. D., on which as yet we have expressed no opinion. We propose now to let the Boston Journal speak for us.—Ed. Buff. Med. Jour.

The author informs us (needlessly, by the way) that he "dissents from the views of writers in general," and does not regard cholera infantum as a disease *sui generis;* that moreover, it is not peculiar to this country, but is, in fact, identical with Asiatic or epidemic cholera; age, only, producing any difference in the phenomena; and "that the disease is essentially a scorbutic affection." We hardly dare to intimate that we have seen the diseases or rather the disease (*scorbutico-infantico-asiatico-cholera*, may we venture to name it?) here referred to, and that we fail, even after reading the collated information spread over several pages by M. L. Knapp, M. D., to see the analogy—the *vinculum*—which so closely unites them. However, our vision must be affected, we will conclude, by our "cloister" life; how fortunate for us, for the world, that such thrilling trumpet-tones, *occasionally*, ring outside our narrow walls! We should like to ask one question; and that is, whether it is not likely that scorbutus existed long before cholera maligna was ever heard of? And if so why did not the latter appear sooner, being so sure a consequence (Knapp) and sequel of the former—and patients with scorbutic affections being so constantly found?

On page 71 of this essay, it is stated that it is the writer's own conviction "that if all breeding and nursing women could have plenty and variety of good animal and vegetable food; were enjoined to indulge in the free use of oranges, lemons, apples, and all kinds of fruits, jellies, *pickles* and *salads* (italics are ours); and infants at the breast were allowed, in addition to the rich vegetable milk emulsion which such a dietary on the part of the mothers would afford them, to suck oranges and roasted apples, and to have lemonade, *vinegarade*, (our italics) &c., as freely as their pantomime inclinations seem to demand, and this course adopted early and persisted in through infancy and early childhood, cholera infantum would be nearly banished from the catalogue of human diseases." These judicious recommendations must, we think, be eagerly and gratefully received.

Dr. Knapp was told by a prominent physician of Cincinnati, that "he (the physician) had known an instance of a child being cured of cholera infantum by eating freely of ripe currants."—(p. 71) The *dictum* of the physician in this instance does not, by any means, prove the currants remedial. The child got well *after* them, very likely. Was nothing else done previously? *Was* the case one of cholera infantum, and if so, of any severity? An affirmative answer to these questions will only make it the more certain that the recovery was not a "cure" by currants, but took place through other influences.—"Blackberry jelly and cordial are popular remedies."—(p. 71.) Yes, and so they are professional ones; a capital astringent is blackberry root tea, also, to our knowledge.—(Records from the "Cloister.")

We are not yet brought to the admission, despite the "twenty years' experience," &c., of this writer, that scorbutus is the *primum mobile* of all the choleraic diseases, and that "cholera infantum is infantile scurvy."—(p. 66.) The subject, in certain of its relative aspects, is worthy of attention; no doubt ill-nourished and scorbutic individuals are fit subjects for cholera, be they infants or adults; but the unfounded assertions and deductions observable throughout this pamphlet hardly merit consideration, when we reflect upon the manner in which any important subject should be investigated.

We know but little of the Western institutions to which this writer has held certain relations, but we can hardly help thinking that those therein instructed will be quite as much edified as formerly—rather more so, indeed, by the present condition of those relations, as we remark that M. L. Knapp, M. D., is now "*late*" Professor, &c.—*Boston Med. and Surg. Jour.*

